# Genome-wide analysis and characterization of TPD1 family proteins in pearl millet (Cenchrus americanus): Insights into reproductive regulation and phytohormone responses

**DOI:** 10.1371/journal.pone.0318196

**Published:** 2025-01-27

**Authors:** Zainab M. Almutairi

**Affiliations:** Department of Biology, College of Science and Humanities in Al-Kharj, Prince Sattam bin Abdulaziz University, Al-kharj, Saudi Arabia; Central South University of Forestry and Technology, CHINA

## Abstract

The Tapetum Determinant 1 (TPD1) family proteins are known to play a crucial role in the regulation of reproduction in plants, including *Cenchrus americanus* (pearl millet). However, members of TPD1 family proteins have not been fully identified. The current study aims to identify and characterize the TPD1 family proteins in *Cenchrus americanus* (L.) Morrone. Seven transmembrane proteins (from 127 to 172 aa) comprising TPD1 domain were identified via genome-wide mining. Analysis of gene expression during developmental stages revealed high expression of four *CaTPD1s* in reproductive organs. Treatment with phytohormones showed that the expression of *CaTPD1s* was repressed by hormone treatments except *CaTPD1_Ch4*.*1* and *CaTPD1_Ch4*.*3* which are highly expressed in response to brassinolide and auxin, respectively. Screening of *cis*-elements in the promoter of *CaTPD1s* revealed various *cis*-elements related to phytohormone regulation, wound response, abiotic stress defense, and light response. The phylogenetic tree revealed distinct clustering of CaTPD1_Ch6 and CaTPD1_Ch5 among the other CaTPD1s, which revealed close relationships with the orthologs from Arabidopsis and rice that are known to have a critical role in tapetum development and pollen and ovule production. Hence, this study affirms the role of the *CaTPD1s* genes in the growth and reproduction during pearl millet developmental stages.

## 1. Introduction

The Tapetum Determinant 1 (TPD1) proteins are multiple small proteins that have been found to share a conserved TPD1 domain in the plant genome. The first identified TPD1 member is a transcription factor known to control tapetum development, a somatic cell layer around male meiocytes in higher plants [1]. The normal growth of the tapetum is necessary for the male meiotic cell cycle. Defects in tapetum development can cause disruptions in the expression of genes involved in male reproduction [2].

Tapetum determinant 1 protein is known to regulate the differentiation of floral organs, pollination, maturation of seeds, embryogenesis, and cell fate determination and patterning by interaction as a ligand with its receptors, Extra Microsporocytes1 (EMS1) and Somatic Embryogenesis Receptor-Like Kinases 1 and 2 (SERK1 and 2; [3]. These three proteins form the EMS1-TPD1-SERK1/2 complex that activates Brassinazole Resistant 1 (BZR1) and Brassinosteroid Insensitive 1 EMS Suppressor 1 (BES1), which are essential transcription factors in the brassinosteroids signaling pathway during tapetum development [4].

Yang et al. (2005) reported that TPD1 protein, in coordination with EMS1/Extra Sporogenous Cells (EXS), regulates apoptotic floral organ death in the Arabidopsis tapetum and carpel. The programmed death of the tapetum cells, which is regulated by Tapetum Degradation Retardation (TDR) and Persistent Tapetal Cell 1 (PTC1), is controlled by TPD1 after the completion of pollination. Furthermore, TPD1 has been demonstrated to have a crucial function in megaspore mother cell proliferation through the auxin signaling pathway during ovule production [5].

Despite that the role of Arabidopsis TPD1 protein is well clarified, the role of its paralogs is less understood. Sequencing of plant genome revealed four TPD1 proteins in Arabidopsis that appear to be Beta-1,3-N-Acetylglucosaminyltransferase family proteins that include several conserved enzymes from type II transmembrane protein containing uncleaved signal anchor [6].

Multiple paralogs to TPD1 have been identified in cereals and their role during reproduction has been reported. Two TPD1 proteins have been identified in rice, *Tapetum Determinant1-Like A (TDL1A)* and *TDL1B* that are expressed in anther during meiosis. However, in the ovule, only *OsTDL1A* is expressed [7]. The ortholog of rice TDL1A in maize called Multiple Archesporial Cells 1 (MAC1), has been identified to have a role during male reproductive organs development [8]. Diploid banana comprised four *TPD1* genes which one of them is found to has a role in pollen and fruit development [9].

Pearl millet (*Cenchrus americanus*) is a C4 cereal crop that is highly tolerant to arid and semi-arid conditions, making it a crucial source of nutrition and fodder in areas where other crops struggle to grow [2]. However, our understanding of the molecular mechanisms governing its reproductive processes, particularly involving the TPD1 protein family, is limited. Previous studies on TPD1 proteins have predominantly focused on model C3 plants like Arabidopsis and rice, which do not capture the unique physiological adaptations of C4 plants like pearl millet. This study aims to address these gaps by providing a genome-wide analysis and characterization of TPD1 proteins in pearl millet, contributing to a broader understanding of the reproductive regulation in C4 species and their response to phytohormone signaling under arid conditions [4].

The reproduction regulation mechanism appears to be different from C3 and C4 cereals, due to the differences in the photosynthesis physiology. In an attempt to identify the TPD1 family proteins in C4 cereals, this study aims to screen the genome of the C4 plant, pearl millet (*Cenchrus americanus* (L.) Morrone) for TPD1 family members by genome-wide analysis. Identification of TPD1 family proteins in pearl millet can aid in understanding the role of these proteins in the regulation of growth and reproduction of C4 cereals.This study hypothesizes that the TPD1 family proteins in pearl millet play a critical role in regulating reproductive processes and phytohormone responses, which are key to the plant’s adaptation to environmental stresses.

## 2. Materials and methods

### 2.1 Database sequence retrieval and plant materials

The PANTHER database was retrieved for TPD1 domain number PTHR33184:SF5 limited to the rice genome. The rice *TPD1* protein sequences were downloaded from Ensembl genome browser 110 [10] and used as queries in the Whole Genome Shotgun Contigs in the NCBI in *C*. *Americanus* genome to find the hypothetical sequence of TPD1 in the *C*. *americanus* genome using tBLASTn tool. For each hypothetical TPD1 protein found, the genomic sequences were downloaded and used to design PCR primers to amplify cDNAs for each TPD1 gene using Primer3 tool (https://primer3.ut.ee/). The primer pairs successfully used to amplify the cDNAs of *CaTPD1* are shown in S1 Table in S1 File. The plant material used in this study was *C*. *americanus variety #1316* obtained from the Saudi Centre of Genetic Resources. Seeds of this specific variety were used for all experiments in this study.

### 2.2 cDNA sequencing and characterization of *CaTPD1s*

Seeds of millets were sterilized in 2% (v/v) NaOCl, for 18 min, rinsed with deionized water, and grown in a Petri dish for three days. Extraction of RNA from 3-day-old seedlings was performed by PureLink™ Plant RNA reagent (Invitrogen) following the manufacturer protocol. The *CaTPD1* cDNAs were amplified using BIO-RAD iScript™ cDNA Synthesis Kit. The PCR experiment was conducted with PCR master mix from Promega using cycling of 98°C for 3 min; then 32 cycles each of 20 s at 94°C, 25 s at 56°C, and 46 s at 72°C; and finally 12 min at 72°C. PCR fragments with the correct size were sequenced with *BigDye* Terminator v3.1 according to Sanger et al. (1977) [11] method.

The obtained cDNA sequences were translated into protein using NCBI open reading frame finder tool, and protein domains were found by the InterPro (https://www.ebi.ac.uk/interpro/). The isoelectric point and molecular weight of the CaTPD1 protein were subsequently calculated using Compute pI/Mw tool in Expasy server. The exon/ intron structure of the cDNAs was illustrated via the Gene Structure Server [12] with the corresponding coding and genomic sequences for each *TDP1* gene. The tertiary structures of the deduced CaTPD1 proteins were modeled using the SWISS-MODEL (https://swissmodel.expasy.org/). Subcellular localization of the translated CaTPD1 proteins was predicted by the DeepLoc-1.0 (dtu.dk). The position of the signal peptide and the transmembrane helices were predicted using the MEMSAT-SVM on the PSIPRED server [13]. Topology of transmembrane residues in CaTPD1 proteins was obtained by CCTOP method [14]. Motif in CaTPD1 protein sequences was screened by the MEME web server (http://meme-suite.org/). A protein-protein interaction was modeled by the STRING (https://string-db.org) limited by *Arabidopsis thaliana* proteins. Gene ontology prediction for CaTPD1 proteins was performed by FFPred 3 tool in PSIPRED server.

### 2.3 Phylogenetic analysis

Sequence alignment of CaTPD1 proteins was carried out using Clustal Omega software to investigate conservation among CaTPD1 proteins. To conduct phylogenetic analysis; 13 TPD1 proteins from rice and seven TPD1 proteins from Arabidopsis were downloaded from Ensembl genome browser 110 [10], and aligned with the CaTPD1 proteins obtained by this study using MEGA 11 software [15]. Then, the phylogenetic tree was built by maximum likelihood method [16] with interior branch tests of 1000 replicates.

### 2.4 *Cis*-regulatory analysis

The putative promoter sequences for *CaTDP1s* were screened for *cis*-elements by retrieving the sequence 1.5 kb upstream from the first codon of each *CaTDP1* using the PlantCARE database [17].

### 2.5 Phytohormone treatments and gene expression analysis

The expression of *CaTPD1* in pearl millet organs were analyzed, during various developmental stages and in response to phytohormones. To examine the expression of *CaTPD1*s during germination; a group of seeds were germinated in a Petri dish for 24h, then subjected to RNA extraction. For expression analysis during growth and reproduction, another group of seeds was grown in the field until ripening to collect tissues at different stages, including leaf, root, and stem of a 21-day-old plant, panicle before heading, anther and carpel during flowering, spikelet after ripping and mature seeds. To analyze the expression of *CaTPD1* in response to plant hormones; seven phytohormones were applied to 48h soaked seeds and 5-day-old seedlings separately as follows: the gibberellic acid (GA), auxin (IAA), salicylic acid **(**SA), cytokinin, jasmonic acid (JA), abscisic acid (ABA), and brassinolide (BL). Soaked seeds and seedlings were treated with 30 uM of each hormone for 3 h, separately, then subjected to RNA extraction. Biological replicates were made up of tissues from 4 different plants.

Primers used to analyze the expression of *CaTPD1s* were designed from the obtained cDNA sequences using Primer3 tool (https://primer3.ut.ee/; S2 Table in S1 File). The qRT-PCR reaction was conducted using KAPA SYBR® FAST qPCR Master Mix. Each reaction was repeated at least three times using the different biological replicates. The delta-delta Ct (ddCt) value was calculated by normalization of *CaTPD1s* cycles to threshold (Ct) values to the reference gene, Elongation factor 1 α Ct values. The expression of *CaTPD1_CH3* in the root tissue was used as a calibrator for the seven *CaTPD1s* in all examined samples. Thereafter, 2^-ddCt^ was calculated as fold-change for *CaTPD1s* expression according to Livak and Schmittgen’s method (2001) [18]. All experiments, including RNA extraction, cDNA synthesis, and qRT-PCR analysis, were conducted with three biological replicates. Each replicate consisted of tissues collected from four different plants to minimize any variability associated with individual specimens.

## 3. Results

### 3.1 *CaTPD1s* Sequence characterization

Seven members of TDP1 family were found in *C*. *americanus* genome and the sequenced cDNAs ranged from 372–522 bp, containing from 1–3 exons as shown in (Table 1, S1 Fig in S1 File). The *CaTPD1* cDNA sequences were deposited in NCBI under the accession numbers: ON040905, OQ731689, OQ943818, OQ943819, OQ943820, OQ943821 and OQ943822. These seven genes were mapped on chromosome number 2, 3, 4, 5 and 6 of *C*. *americanus* genome (three members were mapped on chromosome 4) and named based on their chromosome number. The length of the deduced CaTPD1 proteins ranged from 127–172 aa. All deduced CaTPD1 proteins were found to comprise a TDP1 domain in the C-terminal, a single transmembrane helix, and a signal peptide in the N-terminal except CaTPD1_Ch5 that does not comprise signal peptide (S2 Fig in S1 File). The length of TDP1 domain in the deduced CaTPD1 proteins ranged from 105–150 aa.

**Table 1:** Basic information about Tapetum Determinant 1 gene family members in *C*. *americanus*. The position of domain, SignalP, and transmembrane helices are indicated by the number of first and last amino acid that the domain or helix spans.

TPD1s Member	cDNA NCBI#	Length (aa)	TM position	MW (kD)	pI	Exons number	SignalP position (aa)	Chromosome mapping	Strand	Domain position (aa)
TPD1_Ch2	OQ943818	127	2–19	13.32	4.91	2	1–20	Ch2: LKME02052028 93235096.. 93235686	+/+	12–119
TPD1_Ch3	OQ943819	165	11–30	17.16	10.63	1	1–24	Ch3: LKME02052029 99616839.. 99617146	+/-	21–163
TPD1_Ch4.1	ON040905	134	5–25	14.48	5.59	1	1–24	Ch4: LKME02052030 15436313.. 154363537	+/-	15–123
TPD1_Ch4.2	OQ731689	137	2–17	14.71	4.50	2	1–19	Ch4:LKME02052030 154929959.. 154930434	+/+	15–125
TPD1_Ch4.3	OQ943820	123	2–18	13.37	9.40	2	1–22	Ch4:LKME02052030 33289392.. 33289973	+/-	14–119
TPD1_Ch5	OQ943821	153	17–37	15.96	4.11	2	None	Ch5: LKME02052031 99497283.. 99498119	+/+	34–148
TPD1_Ch6	OQ943822	172	7–26	18.08	4.77	3	1–25	Ch6: LKME02052032 68955105.. 68955916	+/+	21–171

aa: Amino acids, TM: Transmembrane helices, MW: Molecular weight of proteins, kD: Kilodalton, pI: Isoelectric point of proteins, SignalP: Signal peptide.

**Fig 1:**
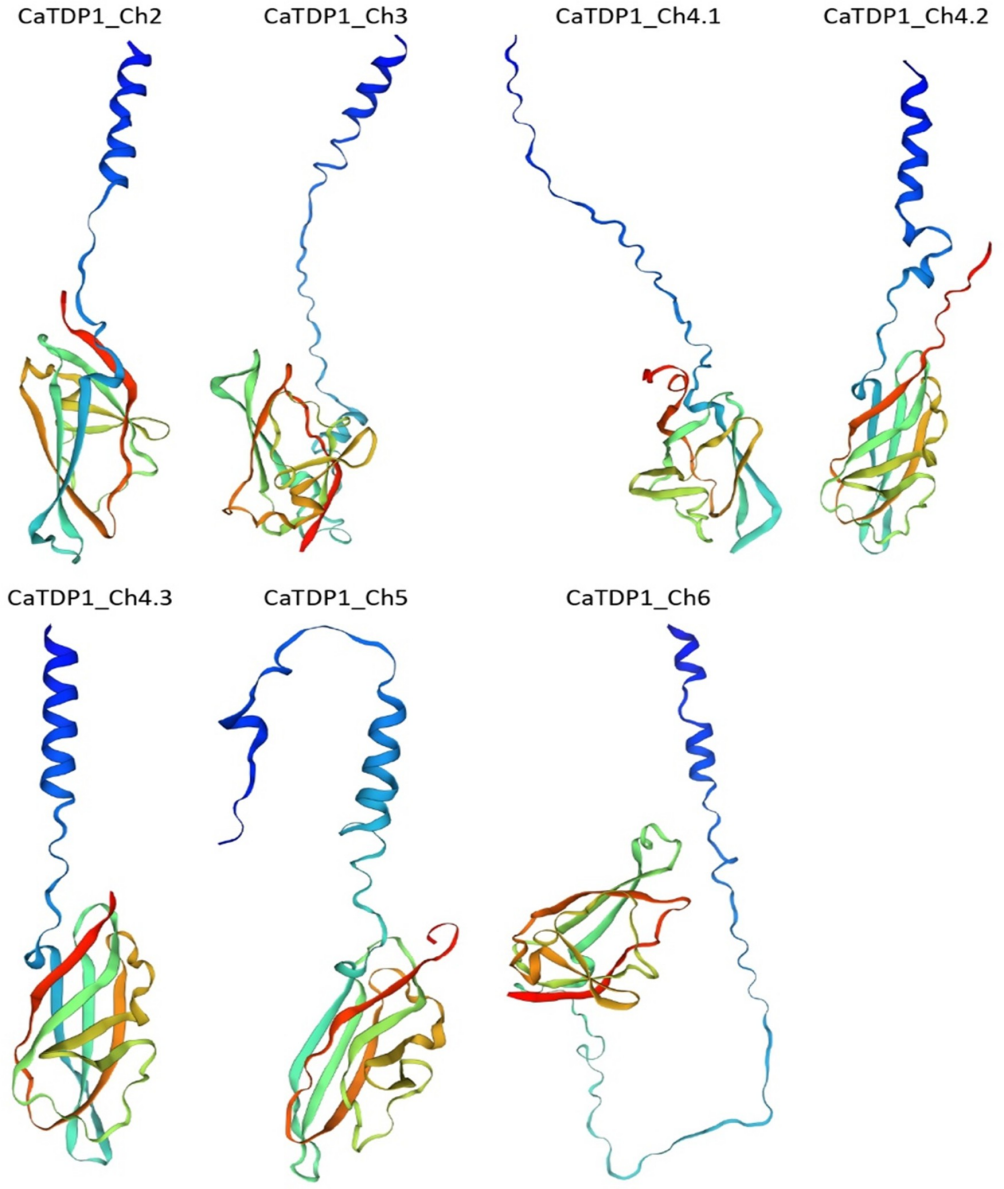
The predicted tertiary structures of the seven Ca*TPD1* proteins from *C*. *americanus* were generated by the SWISS-MODEL analysis. The predicted tertiary structures of the seven CaTPD1 proteins from C. americanus were generated by SWISS-MODEL analysis. Key features include the conserved TPD1 domain across all proteins and the distinct lack of a signal peptide in CaTPD1_Ch5, which also displays a unique topology compared to the other six proteins.

The tertiary structure of CaTPD1 proteins is modeled by the most similar templates in the SWISS-MODEL template library sharing 73.44–93.28% sequence similarity (Fig 1, S3 Table in S1 File). The seven CaTPD1 proteins appeared to share a conserved tertiary structure, despite the different N-terminal of TPD1_Ch5 and TPD1_Ch6 proteins. Subcellular localization showed that six members of CaTPD1 proteins are extracellular proteins with >91% probability, while TPD1_Ch5 is located in the cytoplasm (S4 Table in S1 File). The consensus topology indicated that the C-terminus of CaTPD1 proteins may be oriented outside the membrane, whereas the N-terminus is oriented inside the membrane. However, the topology of TPD1_Ch5 member appeared to be different from the other six CaTPD1 proteins, in which the C-terminus of TPD1_Ch5 protein may be oriented inside the membrane, whereas the N-terminus is oriented outside the membrane (S5 Table in S1 File).

**Fig 2:**
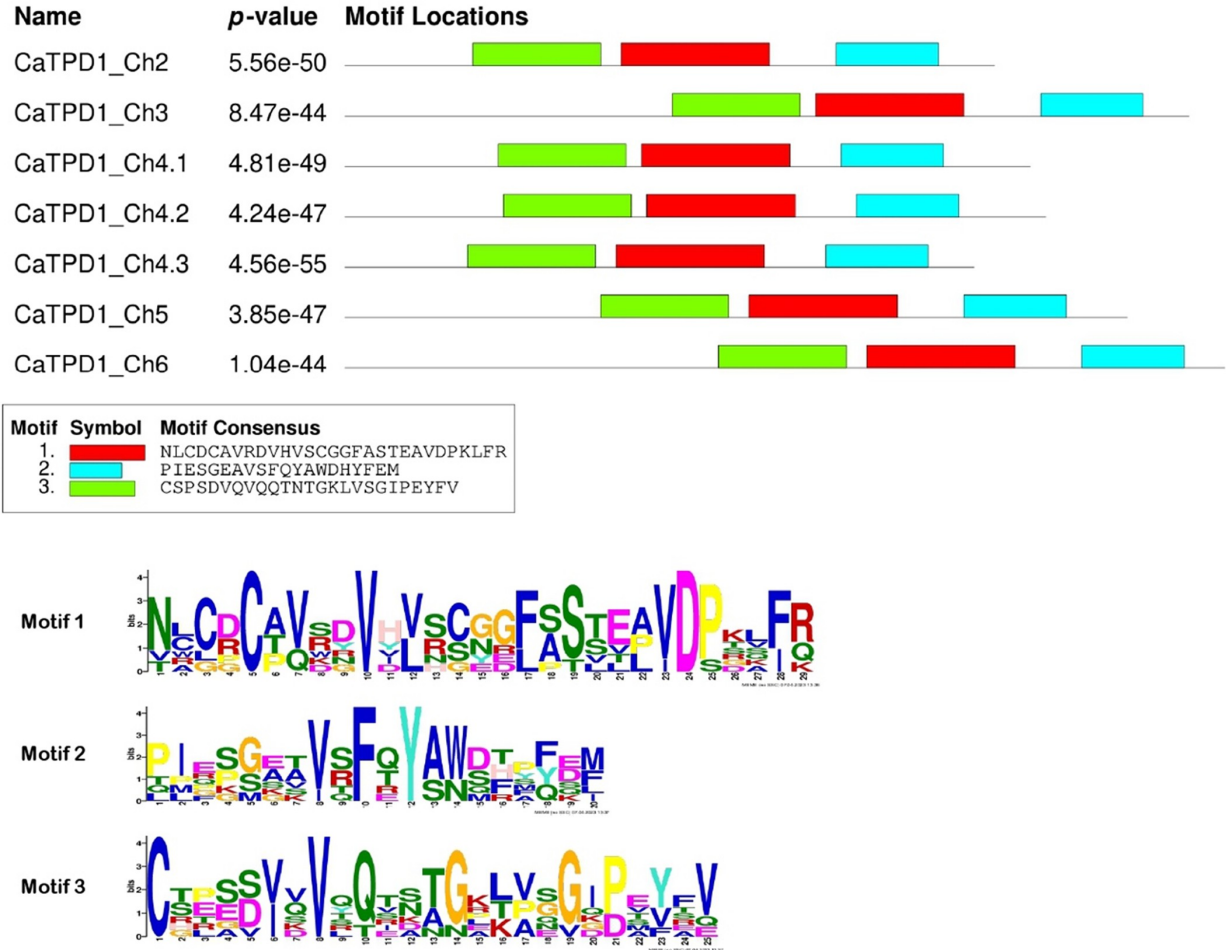
Conserved motif identification among the seven TPD1 proteins in *C*. *americanus* predicted by MEME server. The consensus sequences of three identified motifs represented by different colored boxes are listed below. The sequence logo for each motif is presented below the consensus sequences. The identified motifs for CaTPD1 proteins are depicted with enlarged motif logos for better clarity. The sequences of the three motifs are as follows: Motif 1: AGCTAG…, Motif 2: TCGAAG…, Motif 3: GATCTA… .

Three motifs are conserved across the seven CaTPD1 proteins with 20, 25, and 29 aa lengths overlapping with TPD1 domain sequence (Fig 2). Strong conservation was shown in the multiple sequence alignment among all CaTPD1 proteins in the N and C-terminal sequences (S3 Fig in S1 File). Likewise, domain architecture across the seven CaTPD1 proteins revealed strong similarity despite TPD1_Ch5 protein that lacked the signal peptide.

Gene ontology prediction for CaTPD1 proteins by FFPred 3 revealed that some molecular functions and biological processes are shared by all CaTPD1 proteins (S6 Table in S1 File) such as cell surface receptor signaling pathway (GO:0007166), regulation of metabolic process (GO:0019222), transport (GO:0006810), and catalytic activity (GO:0003824 and GO:0005125). Notably, some molecular functions and biological processes appeared to be specific to CaTPD1_Ch5 such as DNA and RNA binding, RNA processing and splicing, ion transport, protein folding and localization, and phosphate-containing compound metabolic process. However, cellular component predictions by FFPred 3 tool revealed that all CaTPD1 proteins found in the endoplasmic reticulum (GO:0005783) and plasma membrane (GO:0005886) except CaTPD1_Ch5 that found in mitochondrion (GO:0005739). Defense response (GO:0006952) appeared to be specific to CaTPD1_Ch6, while lipid metabolic process (GO:0006629), sensory perception (GO:0007600) and cellular lipid metabolic process (GO:0044255) appeared to be specific to CaTPD1_Ch2. The three proteins; CaTPD1_Ch3, CaTPD1_Ch5, and CaTPD1_Ch6 appeared to be involved in the regulation of transcription (GO:1903506).

### 3.2 Protein-protein interaction

**Fig 3:**
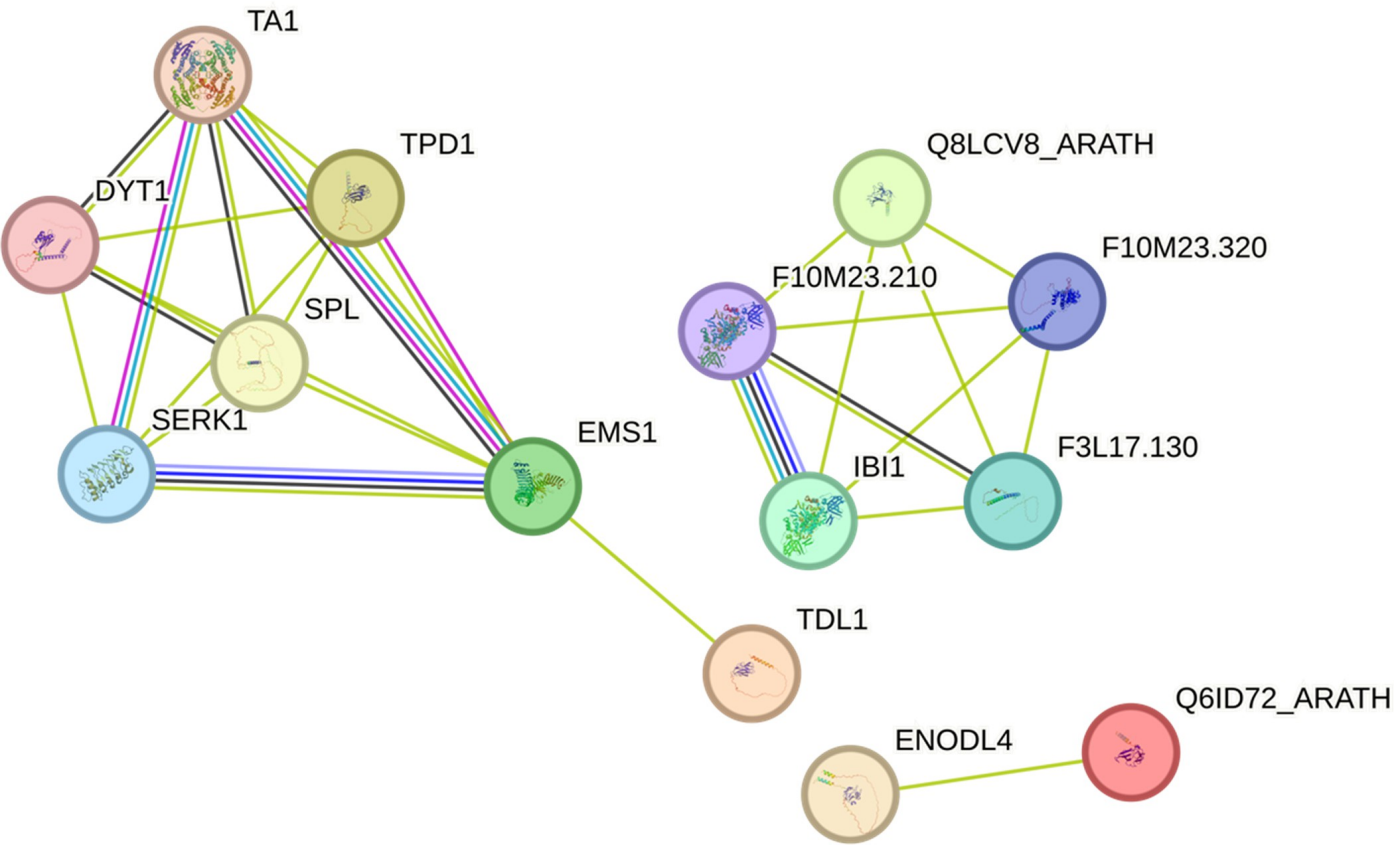
Protein-protein interaction for the seven *CaTPD1* proteins from *C*. *americanus* by String database. The Arabidopsis orthologs proteins as follows: TPD1 (AT4G24972), TDL1 (AT1G32583), and two Beta-1,3-N-Acetylglucosaminyltransferase family proteins; Q6ID72_ARATH (AT4G32110) and Q8LCV8_ARATH (AT4G32105). The shown network proteins are: DYT1, ENODL4, EMS1, SERK1, F10M23.210: Aspartate—tRNA ligase 1; F10M23.320: RNI-like superfamily protein, F3L17.130: High chlorophyll fluorescence 153, IBI1, SPL, and TA1. The network interactions between CaTPD1 proteins and Arabidopsis orthologs are shown. Solid lines indicate direct interactions, while dashed lines represent predicted interactions. The line thickness indicates the strength of data support.

String database revealed 32.7–53.3% identity between the CaTPD1 proteins and four orthologs proteins from Arabidopsis as follows; AT4G24972, AT1G32583, AT4G32110, and AT4G32105 (Fig 3). The Arabidopsis TPD1 protein #AT4G24972, which the most similar ortholog to CaTPD1_Ch5, has an interaction network with EMS1, SERK1, Dysfunctional tapetum 1 (DYT1), Short-chain dehydrogenase reductase ATA1 (TA1), and Sporocyteless (SPL). This network interacts as a second shell interaction with the Arabidopsis TPD1 protein homolog 1 (TDL1; AT1G32583), the closest ortholog to CaTPD1_Ch6. Two Arabidopsis beta-1,3-N-acetylglucosaminyltransferase family proteins appeared to be close ortholog to CaTPD1_Ch4.1; Q6ID72_ARATH (AT4G32110) and Q8LCV8_ARATH (AT4G32105). AT4G32105 protein formed another network including interaction with Aspartate-tRNA ligase 1 and 2 (F10M23.210 and IBI1), RNI-like superfamily protein (F10M23.320), High chlorophyll fluorescence 153 (F3L17.130). Additionally, the Arabidopsis protein AT4G32110 interacts with Early nodulin-like protein 4 (ENODL4). However, string analysis revealed no orthologs in Arabidopsis for the four CaTPD1 proteins; CaTPD1_Ch2, CaTPD1_Ch3, CaTPD1_Ch4.2, and CaTPD1_Ch4.3.

### 3.3 Phylogenetic tree

**Fig 4:**
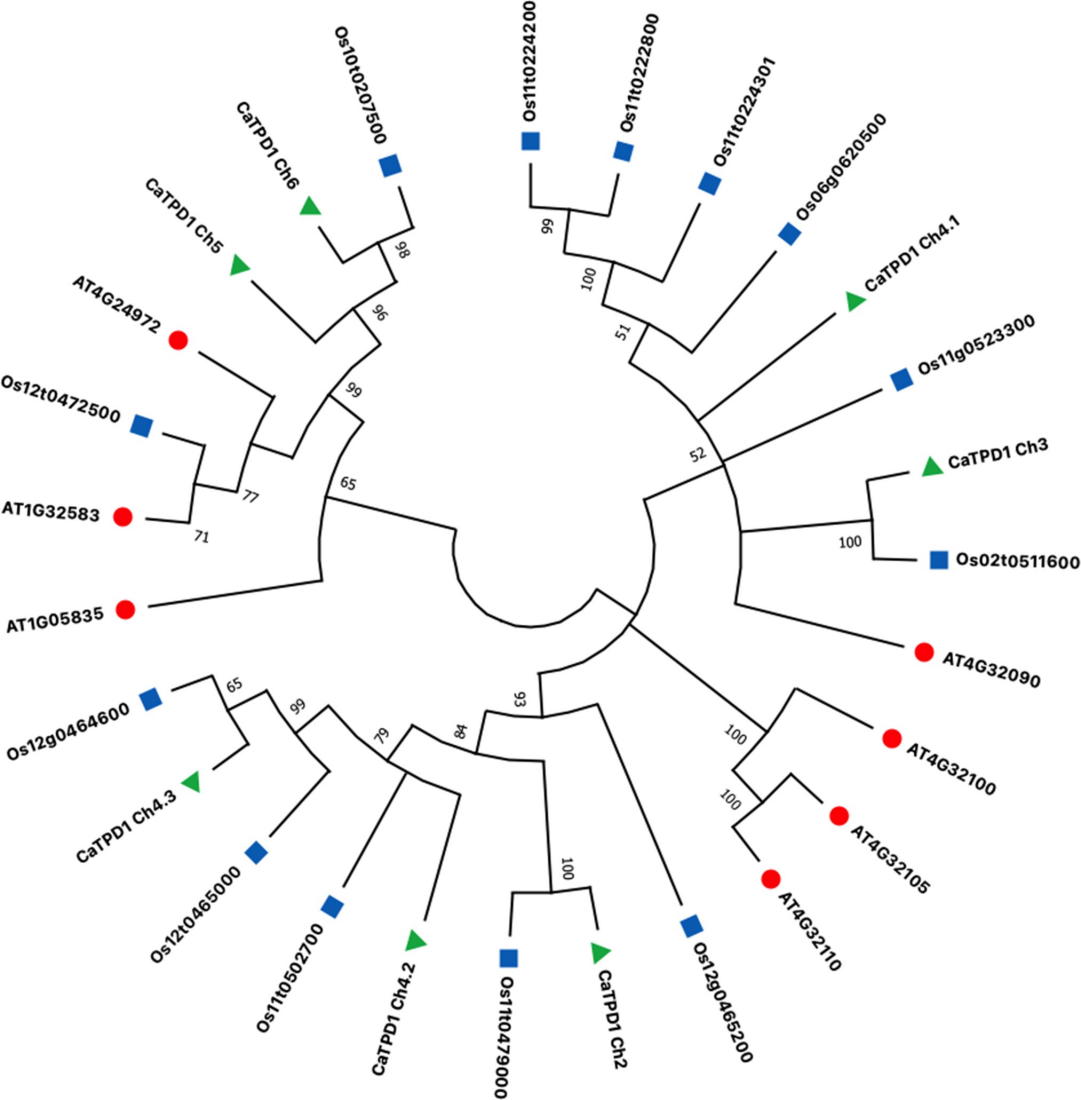
Phylogenetic analysis of the seven CaTPD1 proteins family in *C*. *americanus* with 13 TPD1 proteins from rice and seven TPD1 proteins from Arabidopsis constructed by maximum likelihood method with 1000 bootstraps using MEGA 11 software. The red circles indicate *Arabidopsis thaliana* TPD1 proteins, the blue squares indicate *Oryza sativa* TPD1 proteins, and the green triangles indicate *C*. *americanus* TPD1 proteins obtained by this study.

Phylogenetic analysis for the seven CaTPD1 proteins of *C*. *americanus* was conducted with seven orthologs TPD1 proteins from Arabidopsis and 13 TPD1 proteins from rice (Fig 4). The constructed tree showed two main clusters, which the first cluster includes CaTPD1_Ch5 and CaTPD1_Ch6 with two rice proteins; Os12t0472500 (OsTDL1A) and Os10t0207500 (OsTDL1B), and two Arabidopsis proteins; TPD1 (AT4G24972), and TDL1 (AT1G32583) with bootstrap support from 65 to 99%. The Arabidopsis’s PhD finger protein; At1g05835, separated as an outgroup of this cluster.

The second main cluster diverged into three subclusters which two of these subclusters included five CaTPD1 proteins. The first subcluster included the *C*. *americanus* proteins CaTPD1_Ch4.1 and CaTPD1_Ch3, with six rice TPD1 proteins, and the Arabidopsis beta-1,3-N-acetylglucosaminyltransferase family proteins; AT4G32090, supported with bootstrap values from 51 to 100%. The second subcluster included only three Arabidopsis beta-1,3-N-acetylglucosaminyltransferase family proteins; AT4G32100, AT4G32110, and AT4G32105 with 100% support values. The third subcluster included the *C*. *americanus* proteins CaTPD1_Ch2, CaTPD1_Ch4.2 and CaTPD1_Ch4.3, with five rice TPD1 proteins, with bootstrap support from 65 to 100%.

### 3.4 Distribution of *cis*-elements in the putative promoter of *CaTPD1*s

**Fig 5:**
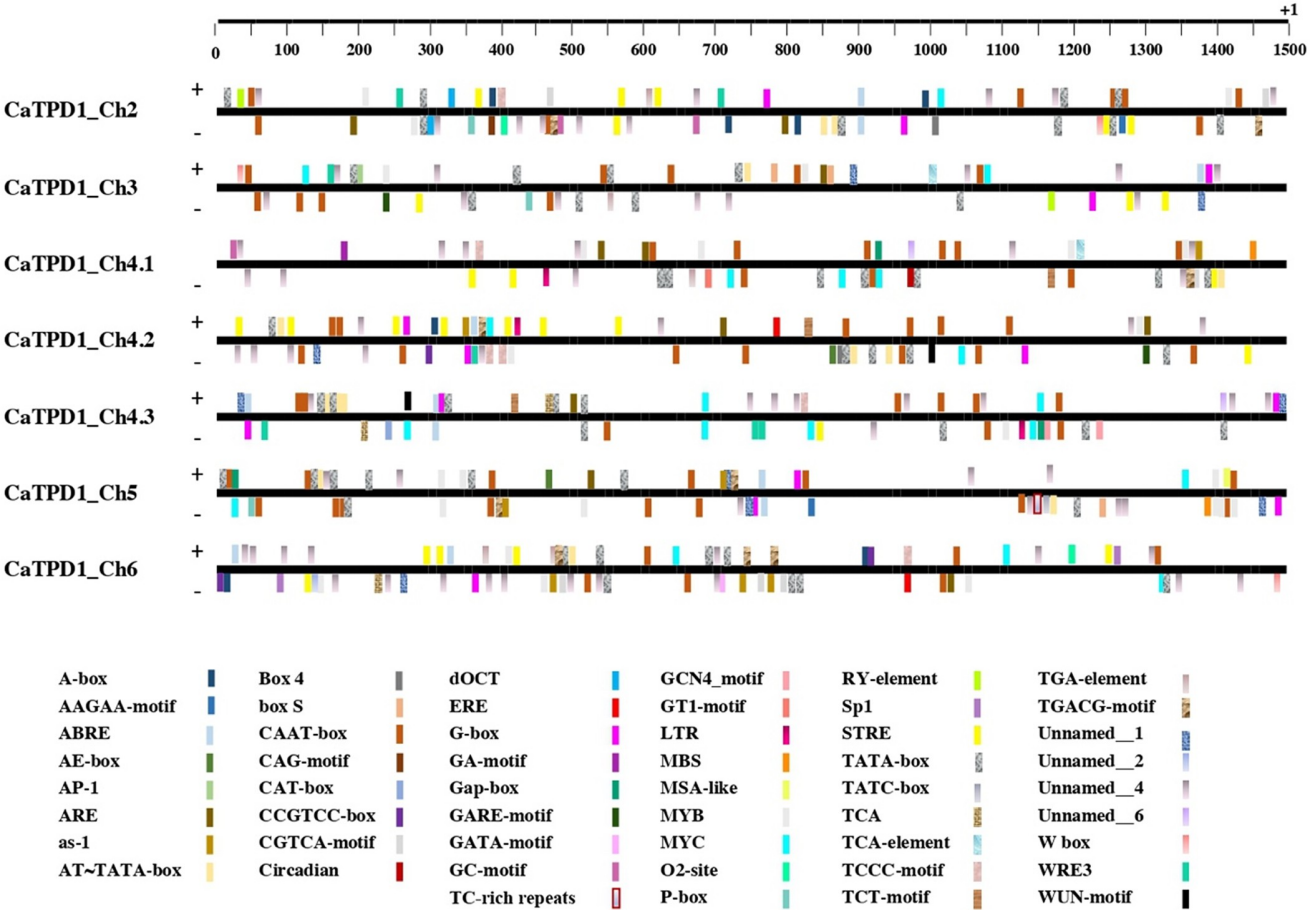
Distribution of *cis*- elements among the putative promoters of the seven Ca*TPD1* genes from *C*. *americanus* predicted by PlantCARE database. The retrieved promoter sequence was 1.5 kp upstream of the start codon. The 1.5 kb 5′-flanking regions of each gene are represented by a line in which the elements above the line are at the plus strand and the elements below the line are at the minus strand. The elements are labeled by different colors and presented below the diagram.

The screening of *cis*-elements in the 1.5 kb putative promoters of *CaTPD1* genes revealed that the promoters of *CaTPD1* genes included binding sites for various light and stress responses and plant hormones (Fig 5, S7 Table in S1 File). Seven regulatory elements appear to be extensively present in all analyzed *CaTPD1* promoters including the core promoter elements; TATA-box and AT~TATA-box, drought-inducibility elements; MYB, and MYC, the regulatory element for anaerobic induction; ARE, the common element in enhancer regions; CAAT-box, and unnamed_4 element (might be responsible for tissue-specific expression). Moreover, various *cis*-elements related to the responses of seven kinds of phytohormones were distributed in some *CaTPD1* promoters including GA (P-box element was found in three *CaTPD1* promoters), ABA (ABRE element was found in six *CaTPD1* promoters), IAA (AAGAA-motif and TGA-element were found in six *CaTPD1* promoters), JA (CGTCA-motif, and TGACG-motif were found in six *CaTPD1* promoters), ethylene (ERE element was found in three *CaTPD1* promoters), cytokinin (as-1 element was found in four *CaTPD1* promoters), and SA (TCA-element, and TCA-motif were found in four *CaTPD1* promoters). Moreover, the analyzed *CaTPD1* promoters contain some stress-responsive elements such as the wound-responsive elements; WRE3, WUN-motif, and W box, drought-responsive element; MBS, anoxic-responsive element; GC-motif, heat-responsive element; STRE, low-temperature-responsive element; LTR, and biotic and abiotic stress responses element; Unnamed__1. Likewise, seven elements related to light response are distributed in all analyzed promotes including; box S, Gap-box, TCT-motif, TCCC-motif, G-box, box 4, and AE-box.

Additionally, other regulatory elements related to cell cycle and metabolism were present in the promoters of more than one *CaTPD1* gene (S7 Table in S1 File). However, fourteen *cis*-elements were found to be specific for the promoter of only one member of *CaTPD1* genes. These included two elements specific to *CaTPD1_CH2* (the light-responsive elements; CAG-motif, and the meristem-specific activation element; dOCT), one element specific to *CaTPD1_CH3* (growth factors or cytokines-responsive element; AP-1), four elements are specific to *CaTPD1_CH4*.*1* (circadian control, light-responsive elements; GA-motif, and GT1-motif and element *cis*-element involved in defense and stress responsiveness; TC-rich repeats), two elements are specific to *CaTPD1_CH4*.*3* (endosperm expression element; GCN4_motif, and the meristem specific activation element; CAT-box), two element are specific to *CaTPD1_CH5* (GA responsiveness elements; TATC-box, and cell cycle regulation element; MSA-like), and three element are specific to *CaTPD1_CH6* (light responsiveness elements; Sp1, and GATA-motif, and Unnamed__2 element that might act as an antisense transcript).

### 3.5 *CaTPD1s* expression patterns

**Fig 6:**
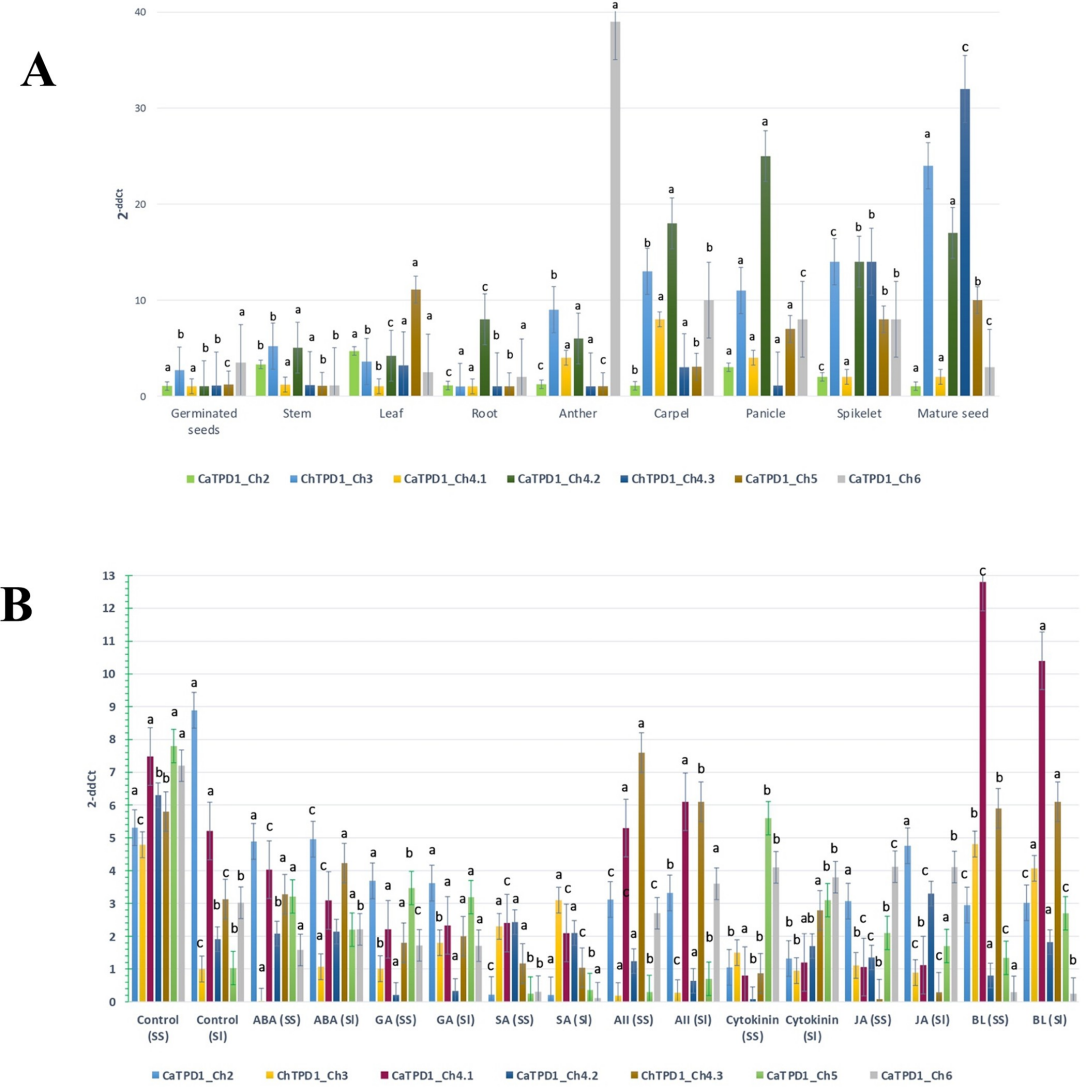
Expression of the seven Ca*TPD1* genes from *C*. *americanus* during different developmental stages and in response to phytohormones as examined by real-time PCR. **A**. The expression of the seven *CaTPD1* genes during germination, vegetative, and reproductive stages; 24h germinated seeds, leaf, root, and stem of 21-day-old plant, anther and carpel during flowering, panicle before heading, spikelet after ripping and mature seeds. **B**. The expression of the seven Ca*TPD1* genes from *C*. *americanus* after treatment with seven phytohormones; gibberellic acid (GA), salicylic acid ***(***SA), auxin (IAA), abscisic acid (ABA), cytokinin, jasmonic acid (JA), and brassinolide (BL). RNA was extracted from 48h soaked seeds (SS) and 5-day-old seedling (SL) after 3h of hormone treatment. The Ct values for the seven Ca*TPD1* genes were normalized concerning Ct value for Elongation factor 1 α of the same sample. Value for each time point represents the mean of three biological replicates. The 2^-ddCt^ value indicates the expression level in the X-axis.

Expression profiles of the seven Ca*TPD1* genes from different *C*. *americanus* tissues during germination, vegetative and reproductive stages revealed high expression (between 11 and 25-fold) for the two genes; *CaTPD1_Ch3*, and *CaTPD1_Ch4*.*2* in the reproductive tissues; carpel, panicle, spikelet, and mature seeds (Fig 6A). The expression of these two genes in the anther were 9, and 6-fold, respectively. However, these two genes showed lower expression in germination and vegetative stages. Likewise, *CaTPD1_Ch4*.*3* showed high expression in spikelet and mature seeds and low expression in other tissues. Notably, *CaTPD1_Ch6* revealed the highest expression level among all examined genes in anther (39-fold) followed by the carpel, panicle, and spikelet (10, 8, and 8-fold, respectively), and low expression in other tissues. In contrast, the highest expression of *CaTPD1_Ch5* was 11-fold in the leaf, followed by mature seeds, spikelet, and panicle (10, 8, and 7-fold, respectively).

The expression of the seven Ca*TPD1* genes was analyzed in response to seven phytohormones in soaked seeds and seedlings with respect to the expression in untreated seeds and seedlings. The difference in the expression level of less than 1-fold was not considered activation nor repression. The seven Ca*TPD1* genes were repressed by the seven phytohormones in soaked seeds and seedlings except for a slight activation in seedling for some Ca*TPD1* genes. Despite that, BL caused a notable increase of the expression of *CaTPD1_Ch4*.*1* in both soaked seeds and seedlings (12.8 and 10.4-fold, respectively) in comparison with *CaTPD1_Ch4*.*1* expression in control (7.8-fold in soaked seeds and 5.21-fold in seedlings). Likewise, IAA induced the expression of *CaTPD1_Ch4*.*3* in both soaked seeds and seedlings (7.6 and 6.1-fold, respectively), in comparison with the relative control which was in soaked seeds 7.8-fold, and in seedlings 5.21-fold (Fig 6B).

## 4. Discussion

The Tapetum determinant 1 family members are signaling proteins, embedded in the cellular membranes and known to play an important role in plant reproduction [1,19]. Seven members of TPD1 family are found in the Arabidopsis [19], and about 13 TPD1 proteins are distributed in the rice genome (Sakai et al., 2013 [20]). However, the function of TPD1 family members is clarified for a limited number of TPD1 family proteins [3,5,7]. We therefore identified seven members of TPD1 family proteins in *C*. *americanus* by genome wide-mining and gene expression profiling. The investigated CaTPD1s were small transmembrane proteins that shared conserved motifs and tertiary structures. Despite this strong conservation, these CaTPD1 proteins revealed different expression patterns in response to phytohormone treatments and distinct clustering with their orthologs from Arabidopsis and rice in the phylogenetic tree. The GO analysis revealed functions such as regulation of metabolic processes and catalytic activity, which are directly linked to gamete development and maturation in pearl millet. These biological processes are crucial for ensuring successful reproduction, particularly under environmental stress conditions. Likewise, gene ontology and protein-protein interaction analysis indicated different roles for some TPD1 family members.

Interestingly, CaTPD1_Ch5 revealed different transmembrane topology and subcellular localization, and lacked the signal peptide, indicating less conservation among the other CaTPD1 proteins. However, CaTPD1_Ch5 protein revealed close phylogenetic relationships with its paralog, CaTPD1_Ch6, and with the previously characterized TPD1 proteins of Arabidopsis and rice including two orthologs from rice; Os12t0472500 (OsTDL1A) and Os10t0207500 (OsTDL1B) and three orthologs from Arabidopsis; TPD1 (AT4G24972), TDL1 (AT1G32583), and At1g05835. The rice OsTDL1A and OsTDL1B are identified to be involved in rice pollen development, while OsTDL1A is involved also in ovule development [7]. Although the two proteins; CaTPD1_Ch5 and CaTPD1_Ch6 clustered distantly from the other five *CaTPD1s* in the phylogenetic tree, the expression of *CaTPD1s* showed that the seven genes were generally expressed in reproductive organs more than in root, stem, and leaf during vegetative stage. However, some exceptions such as *CaTPD1_Ch2* showed no differences in expression during germination, vegetative and reproductive stages. Generally, the expression of *CaTPD1s* shown by our study is in accordance with the available data about the expression of *TPD1s* from rice and Arabidopsis [5,7].

Protein-protein interaction revealed a network consisting of the Arabidopsis proteins, TPD1 and TDL1, with five proteins involved in anther development and cell fate determination; SERK1, EMS1, DYT1, SPL, and TA1. This interaction between SERK1, EMS1, DYT1, TA1, and TPD1 was reported by previous studies to be essential for tapetum development, pollen differentiation, and cell fate determination [21]. The transcriptional regulator of sporocyte development, SPL, is required for ovule and embryo sac development [22].

Five CaTPD1 proteins; CaTPD1_Ch4.1, CaTPD1_Ch4.2, CaTPD1_Ch4.3, CaTPD1_Ch2, and CaTPD1_Ch3, clustered in the phylogenetic tree with 11 uncharacterized TPD1 proteins of rice and four Arabidopsis beta-1,3-N-Acetylglucosaminyltransferase family proteins. Despite that the role of these orthologs proteins of Arabidopsis has not been reported yet, the Arabidopsis protein #AT4G32105 (the ortholog for CaTPD1_Ch4.1) interacts with four proteins required for various processes in plant development and stress defense. This network included Aspartate—tRNA ligase 1 and 2 that involved pathogen defense, RNI-like superfamily protein that involved in protein catabolic process, and High chlorophyll fluorescence 153. Another Arabidopsis protein, AT4G32110, appeared to interact separately with Early nodulin-like protein 4 (ENODL4) involved in electron transfer activity. These different interaction networks for the CaTPD1 orthologs indicated the different roles of each member of the TPD1 family.

Beta-1,3-N-acetylglucosaminyltransferase family protein includes diverse membrane galactosyltransferase enzymes, involved in protein amino acid glycosylation. Homology of five CaTPD1 proteins (CaTPD1_Ch4.1, CaTPD1_Ch4.2, CaTPD1_Ch4.3, CaTPD1_Ch2, and CaTPD1_Ch3) with beta-1,3-N-acetylglucosaminyltransferase family proteins from Arabidopsis indicated for the role of these five CaTPD1s in glycosylation of the extracellular and transmembrane proteins. These five CaTPD1 genes expressed in reproductive organs more than in vegetative stages except *CaTPD1_Ch2* that showed similar expression levels during all developmental stages. Gene ontology prediction for CaTPD1 proteins revealed that sensory perception and cellular lipid metabolic process are specific to CaTPD1_Ch2 protein.

Gene expression in response to phytohormone treatments and promoter screening for regulatory elements provide evidence of the regulation of *CaTPD1* genes by phytohormones. The activation of *CaTPD1_Ch4*.*1* and *CaTPD1_Ch4*.*3* by BL and AII, respectively, agreed with the reported role of TPD1 proteins in the activation of the transcription factors responsible for brassinosteroids and auxin signaling pathways during plant reproduction [3,4]. Otherwise, gene ontology revealed that besides the conserved function for all CaTPD1 proteins in cell signaling, transport, and catalytic activity; RNA processing and protein folding, and localization are specific for CaTPD1_Ch5, while defense response is specific for CaTPD1_Ch6. The transmembrane proteins generally play a critical role in transport via cellular membranes and participate in the regulation of transcription and protein folding and localization [23]. The *cis*-element screening revealed that drought-responsive elements were found in the seven *CaTPD1* promoters. This might indicate a possible role of *CaTPD1s* in pearl millet adaptation to drought during reproduction. However, some regulation elements related to meristem, endosperm, circadian and cell cycle were found to be specific to the promoter of some *CaTPD1s*, indicating for specific roles for some *CaTPD1* proteins.

Comparative analysis of the TPD1 family in pearl millet reveals both conserved and unique features when compared to C3 species like Arabidopsis and rice. Similarities in domain architecture suggest a conserved role in reproductive regulation across species; however, differences in expression patterns, particularly in response to phytohormones, underscore the potential adaptation of pearl millet to environmental stresses such as drought and salinity (Huang et al., 2016a; Zhao et al., 2008).

The observed high expression of CaTPD1_Ch3 and CaTPD1_Ch4.2 in reproductive tissues implies a critical role for these genes in gametophyte development, which is likely associated with the increased resilience of pearl millet under harsh conditions. The differential response to phytohormones such as brassinolide and auxin highlights the involvement of these proteins in complex hormonal regulation pathways that govern stress response and reproductive development (Xu et al., 2014).

Future work should focus on functional validation of the roles of CaTPD1 genes through gene knockout or overexpression experiments. These functional studies will help establish the direct role of CaTPD1 proteins in regulating reproductive development and stress adaptation in pearl millet.

## 5. Conclusion

In this study, cDNAs of seven members of the TPD1 family were found by genome wide-mining in *C*. *americanus* genome, sequenced and characterized in silico. Gene expression analyses during growth and reproduction and expression in response to phytohormones proved that several members of CaTPD1s are involved in the regulation of development of male and female gametes and embryo development. The combined results of gene expression, protein-protein interaction, gene ontology, phylogenetic relationships, and promoter analysis indicated the involvement of TPD1 family members in various cellular processes that are required during reproduction and embryo development. The present study provided a scientific base for further investigation of the functions of CaTPD1 proteins in C4 cereals growth and reproduction.

In conclusion, we identified and characterized seven members of the TPD1 protein family in *C*. *americanus* through a genome-wide approach. These proteins exhibited distinct expression profiles during different developmental stages and in response to phytohormone treatments, suggesting diverse roles in reproductive development and stress adaptation. The results highlight the importance of CaTPD1 proteins in promoting reproductive success in arid environments, making them potential targets for crop improvement strategies aimed at enhancing resilience in C4 cereals. This study provides a foundation for future functional analyses that may ultimately lead to improved yields and stress tolerance in pearl millet and other economically important crops.

## Supporting information

S1 File (DOCX)
