# Supplementary material for: Genome-wide analysis and characterization of TPD1 family proteins in pearl millet (Cenchrus americanus): Insights into reproductive regulation and phytohormone responses
Source: PLoS One. 2025 Jan 27;20(1):e0318196. doi: 10.1371/journal.pone.0318196 (PMC11771933; doi:10.1371/journal.pone.0318196)
Supplement: S1 File — (DOCX) [file pone.0318196.s001.docx]

**Supplementary materials**

**Table S1:** Primers used for *CaTPD1s* cDNAs synthesis.

| **Gene** | **Forward primer** | **Reverse primer** |
| --- | --- | --- |
| **CATPD1_CH2** | 5’ ATCAGGCGGTACAGATTCGT 3’ | 5’ ACTCGTCAGATGGATCACAGA 3’ |
| **CATPD1_CH3** | 5’ GCTCAGACGGCGTGAATAAG 3’ | 5’ CACTTTGCATCTCAGTCGCA 3’ |
| **CATPD1_CH4.1** | 5’ ACCAAAATCCCCTTCTGGCT 3’ | 5’ TCCTAATGTCGGCGGATTGA 3’ |
| **CATPD1_CH4.2** | 5’ CCCCTTGCATTCCACAAACA 3’ | 5’ CTTAACCTGCGTCGCCTTAG 3’ |
| **CATPD1_CH4.3** | 5’ AGCACCTCGTACAGTTTCTCT 3’ | 5’ GACCCTTCATAAGCCACACAC 3’ |
| **CATPD1_CH5** | 5’ CGTCCTTGTTTGCTCTCGAG 3’ | 5’ GCCCAGCGCCTAAATATCAC 3’ |
| **CATPD1_CH6** | 5’ ACTGTTCTTCTAGCCGCTGA 3’ | 5’ CGACGATGAACAGAGAGCAC 3’ |

**Table S2:** Primers used for *CaTPD1s* gene expression analysis.

| **Gene** | **Forward primer** | **Reverse primer** |
| --- | --- | --- |
| **CATPD1_CH2** | 5’TTCAAGGTACCAGTGGCGAG 3’ | 5’ACGGGCAGAGGTTCTTCAC 3’ |
| **CATPD1_CH3** | 5’ GTCGCCACCACAACCACC 3’ | 5’ GATGATGATGGTGGTGGTGC 3’ |
| **CATPD1_CH4.1** | 5’ GGCACCTGTCATCTGTCTGA 3’ | 5’ GTAATCCTGCGGGCAATCG 3’ |
| **CATPD1_CH4.2** | 5’ CTTTCTGCTCCTCCTTGTGC 3’ | 5’ AGTTCTGCACCATCACCTCA 3’ |
| **CATPD1_CH4.3** | 5’ TTTGTGCTCTTCCTGGTTGC 3’ | 5’ GTCACCTGAAACACCGTGTC 3’ |
| **CATPD1_CH5** | 5’ GACAACGACACGACCACC3’ | 5’AGCAGTCGTTGTCCAGCA 3’ |
| **CATPD1_CH6** | 5’ GAAATGTGCTTCTCTGGTGGT 3’ | 5’ TGAAGAGGAAGACGACGCAT 3’ |
| **Elongation factor 1 α** | 5′GTTACAACCCAGACAAGATTGC 3′ | 5′ TGGACCTCTCAATCGTGTTG 3′ |

**Table S3:** Tertiary structure templates and sequence identity with TPD1 family proteins of in *C. Americanus* modeled by Expasy SWISS-MODEL

| Gene | template | Identity |
| --- | --- | --- |
| CATPD1_CH2 | [A0A811Q6V0.1.A](https://swissmodel.expasy.org/repository/uniprot/A0A811Q6V0?model=AF-A0A811Q6V0-F1-model-v4) | 72.44% |
| CATPD1_CH3 | [A0A368RT28.1.A](https://swissmodel.expasy.org/repository/uniprot/A0A368RT28?model=AF-A0A368RT28-F1-model-v4) | 86.75% |
| CATPD1_CH4.1 | [K3ZME0.1.A](https://swissmodel.expasy.org/repository/uniprot/K3ZME0?model=AF-K3ZME0-F1-model-v4) | 93.28% |
| CATPD1_CH4.2 | [K3ZMH6.1.A](https://swissmodel.expasy.org/repository/uniprot/K3ZMH6?model=AF-K3ZMH6-F1-model-v4) | 85.50% |
| CATPD1_CH4.3 | [A0A2T7EGV0.1.A](https://swissmodel.expasy.org/repository/uniprot/A0A2T7EGV0?model=AF-A0A2T7EGV0-F1-model-v4) | 86.18% |
| CATPD1_CH5 | [A0A3L6TSQ6.1.A](https://swissmodel.expasy.org/repository/uniprot/A0A3L6TSQ6?model=AF-A0A3L6TSQ6-F1-model-v4) | 80.85% |
| CATPD1_CH6 | [A0A811MRP8.1.A](https://swissmodel.expasy.org/repository/uniprot/A0A811MRP8?model=AF-A0A811MRP8-F1-model-v4) | 81.55% |

**Table S4:** Subcellular localization of TPD1 family proteins of in *C. americanus* predicted by DeepLoc-1.0.

| **Entry ID** | **Localization** | **Type** | **Nucleus** | **Cytoplasm** | **Extracellular** | **Mitochondrion** | **Cell membrane** | **Endoplasmic reticulum** | **Plastid** | **Golgi apparatus** | **Lysosome/Vacuole** | **Peroxisome** |
| --- | --- | --- | --- | --- | --- | --- | --- | --- | --- | --- | --- | --- |
| **CaTPD1_Ch2** | Extracellular | Soluble | 0.0003 | 0.0023 | 0.9611 | 0.0012 | 0.002 | 0.0111 | 0.0011 | 0.0 | 0.0209 | 0.0 |
| **ChTPD1_Ch3** | Extracellular | Soluble | 0.0 | 0.0001 | 0.919 | 0.0 | 0.0004 | 0.0066 | 0.0 | 0.0 | 0.074 | 0.0 |
| **CaTPD1_Ch4.1** | Extracellular | Soluble | 0.0 | 0.0 | 0.9547 | 0.0 | 0.0004 | 0.0042 | 0.0 | 0.0 | 0.0407 | 0.0 |
| **CaTPD1_Ch4.2** | Extracellular | Soluble | 0.0 | 0.0 | 0.9907 | 0.0 | 0.0 | 0.0002 | 0.0 | 0.0 | 0.0091 | 0.0 |
| **ChTPD1_Ch4.3** | Extracellular | Soluble | 0.0 | 0.0 | 0.9645 | 0.0 | 0.0 | 0.0003 | 0.0 | 0.0 | 0.0352 | 0.0 |
| **CaTPD1_Ch5** | Cytoplasm | Soluble | 0.1127 | 0.263 | 0.1416 | 0.0358 | 0.0379 | 0.0414 | 0.1024 | 0.0212 | 0.2253 | 0.0187 |
| **CaTPD1_Ch6** | Extracellular | Soluble | 0.0 | 0.0 | 0.964 | 0.0 | 0.0012 | 0.0053 | 0.0 | 0.0002 | 0.0292 | 0.0 |

**Table S5:** Topology of transmembrane residues in TPD1 family proteins in *C. Americanus* predicted by CCTOP method (http://cctop.enzim.ttk.mta.hu)

| **Protein** | **reliability** | **numTM** | **length** | **topology** |
| --- | --- | --- | --- | --- |
| **CATPD1_CH2** | 66 | 1 | 127 | IMMMMMMMMMMMMMMMMMMOOOOOOOOOOOOOOOOOOOOOOOOOOOOOOOOOOOOOOOOOOOOOOOOOOOOOOOOOOOOOOOOOOOOOOOOOOOOOOOOOOOOOOOOOOOOOOOOOOOOOOOOOOOO |
| **CATPD1_CH3** | 67 | 1 | 165 | IIIIIIIIIIMMMMMMMMMMMMMMMMMMMOOOOOOOOOOOOOOOOOOOOOOOOOOOOOOOOOOOOOOOOOOOOOOOOOOOOOOOOOOOOOOOOOOOOOOOOOOOOOOOOOOOOOOOOOOOOOOOOOOOOOOOOOOOOOOOOOOOOOOOOOOOOOOOOOOOOOOOO |
| **CATPD1_CH4.1** | 88 | 1 | 134 | IIIIMMMMMMMMMMMMMMMMMMMMMOOOOOOOOOOOOOOOOOOOOOOOOOOOOOOOOOOOOOOOOOOOOOOOOOOOOOOOOOOOOOOOOOOOOOOOOOOOOOOOOOOOOOOOOOOOOOOOOOOOOOOOOOOOOO |
| **CATPD1_CH4.2** | 77 | 1 | 137 | IMMMMMMMMMMMMMMMMOOOOOOOOOOOOOOOOOOOOOOOOOOOOOOOOOOOOOOOOOOOOOOOOOOOOOOOOOOOOOOOOOOOOOOOOOOOOOOOOOOOOOOOOOOOOOOOOOOOOOOOOOOOOOOOOOOOOOOOO |
| **CATPD1_CH4.3** | 95 | 1 | 123 | IMMMMMMMMMMMMMMMMMOOOOOOOOOOOOOOOOOOOOOOOOOOOOOOOOOOOOOOOOOOOOOOOOOOOOOOOOOOOOOOOOOOOOOOOOOOOOOOOOOOOOOOOOOOOOOOOOOOOOOOOOO |
| **CATPD1_CH5** | 95 | 1 | 153 | OOOOOOOOOOOOOOOOMMMMMMMMMMMMMMMMMMMMMIIIIIIIIIIIIIIIIIIIIIIIIIIIIIIIIIIIIIIIIIIIIIIIIIIIIIIIIIIIIIIIIIIIIIIIIIIIIIIIIIIIIIIIIIIIIIIIIIIIIIIIIIIIIIIIIIIII |
| **CATPD1_CH6** | 76 | 1 | 172 | IIIIIIMMMMMMMMMMMMMMMMMMMMOOOOOOOOOOOOOOOOOOOOOOOOOOOOOOOOOOOOOOOOOOOOOOOOOOOOOOOOOOOOOOOOOOOOOOOOOOOOOOOOOOOOOOOOOOOOOOOOOOOOOOOOOOOOOOOOOOOOOOOOOOOOOOOOOOOOOOOOOOOOOOOOOO |


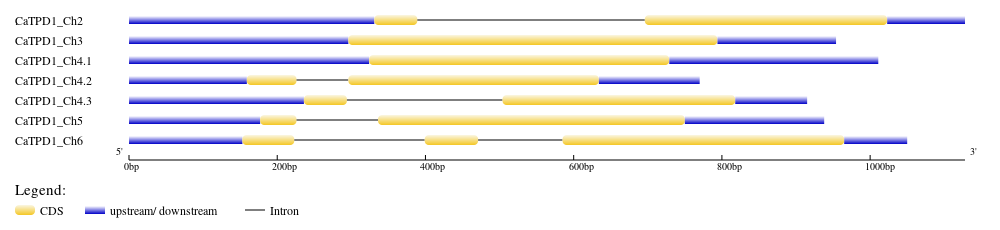


**Fig. S1:** Exon-intron structure for the seven Ca*TPD1* genes from *C. americanus* visualized by Gene Structure Display Server. The genomic sequences for each gene were retrieved from the *C. americanus* genome in NCBI database using the cDNA sequences of each Ca*TPD1* as query.

**
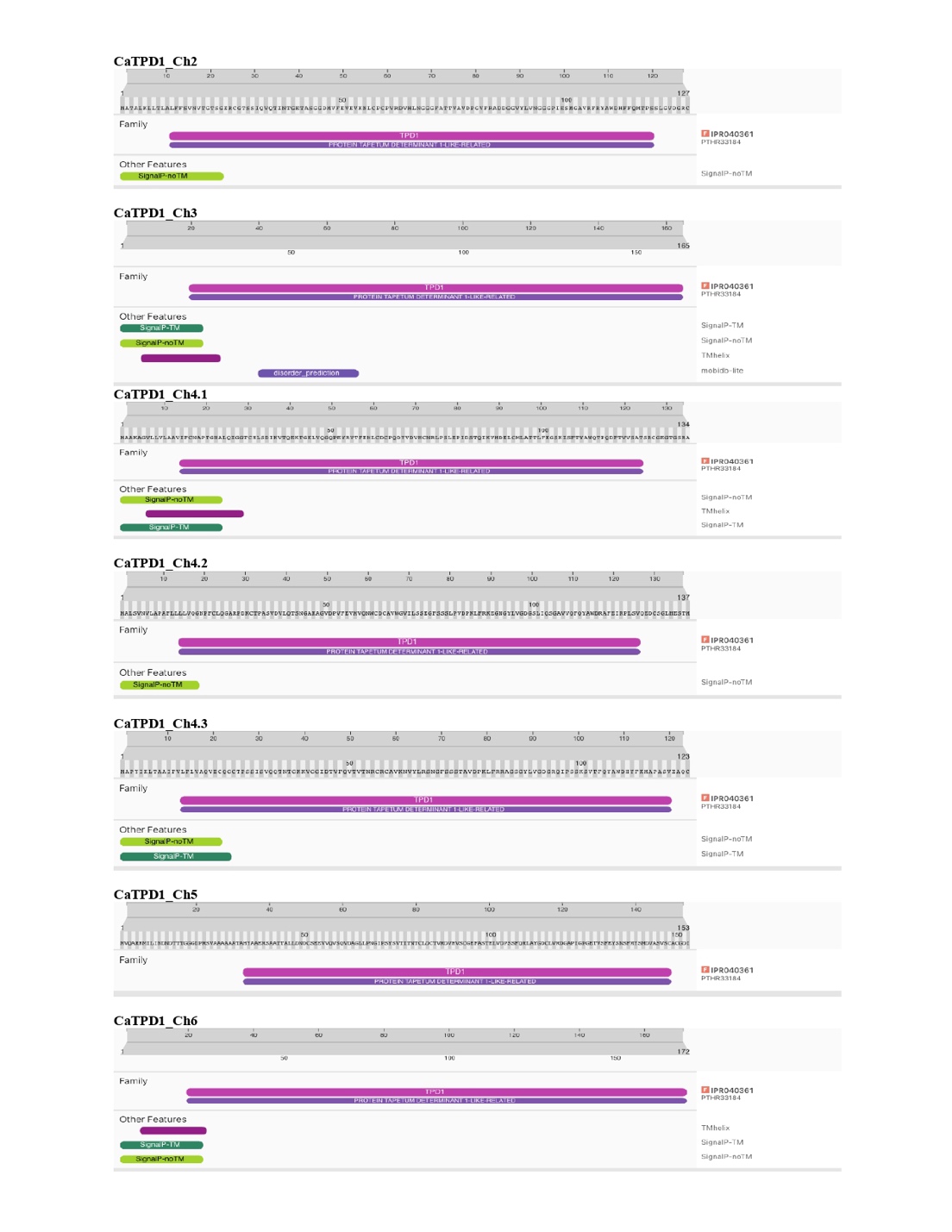
**

**Fig. S2.** Domain architecture in the deduced CaTPD1 proteins from *C. americanus* as predicted by the InterPro.


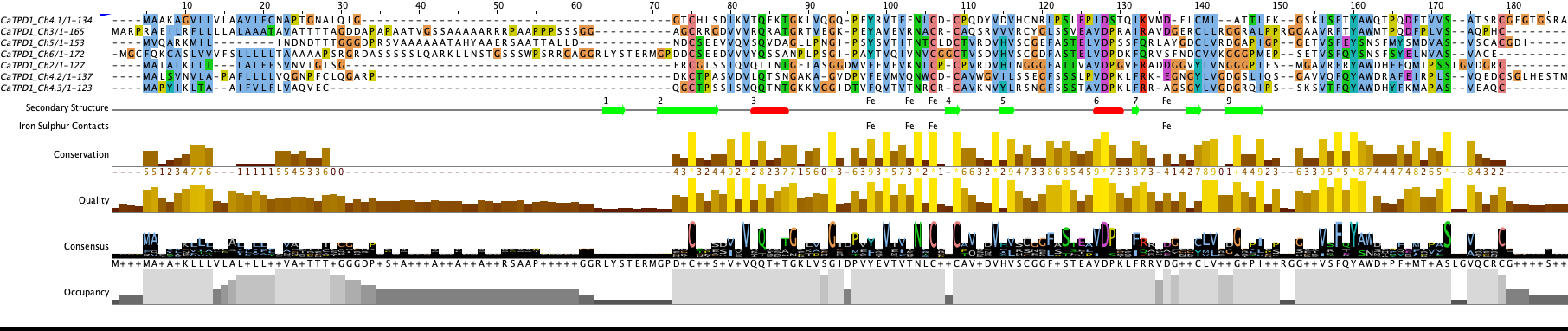


**Fig S3:** Multiple sequence alignments of the seven CaTPD1 proteins from *C. americanus* performed by Clustal Omega software.

**Table S6.** Gene ontology prediction for CaTPD1 proteins by FFPred 3

| **Biological Process Predictions** | | |
| --- | --- | --- |
| **GO term** | **name** | **CaTPD1 proteins** |
| GO:0000375 | RNA splicing, via transesterification reactions | CaTPD1_Ch5 |
| GO:0000398 | mRNA splicing, via spliceosome | CaTPD1_Ch5 |
| GO:0002376 | immune system process | CaTPD1_Ch5 CaTPD1_Ch4.2 CaTPD1_Ch4.1 |
| GO:0002682 | regulation of immune system process | CaTPD1_Ch4.3 CaTPD1_Ch2 |
| GO:0002684 | positive regulation of immune system process | CaTPD1_Ch2 |
| GO:0006351 | transcription, DNA-templated | CaTPD1_Ch5 |
| GO:0006355 | regulation of transcription, DNA-templated | CaTPD1_Ch5 |
| GO:0006396 | RNA processing | CaTPD1_Ch5 |
| GO:0006397 | mRNA processing | CaTPD1_Ch5 |
| GO:0006457 | protein folding | CaTPD1_Ch5 |
| GO:0006508 | proteolysis | CaTPD1_Ch2 |
| GO:0006629 | lipid metabolic process | CaTPD1_Ch2 |
| GO:0006796 | phosphate-containing compound metabolic process | CaTPD1_Ch5 |
| GO:0006810 | transport | CaTPD1_Ch6 CaTPD1_Ch5 CaTPD1_Ch4.3 CaTPD1_Ch4.2 CaTPD1_Ch4.1 CaTPD1_Ch3 CaTPD1_Ch2 |
| GO:0006811 | ion transport | CaTPD1_Ch5 |
| GO:0006812 | cation transport | CaTPD1_Ch5 |
| GO:0006814 | sodium ion transport | CaTPD1_Ch5 |
| GO:0006952 | defense response | CaTPD1_Ch6 |
| GO:0006955 | immune response | CaTPD1_Ch6 |
| GO:0006955 | immune response | CaTPD1_Ch5 |
| GO:0007166 | cell surface receptor signaling pathway | CaTPD1_Ch6 CaTPD1_Ch5 CaTPD1_Ch4.3 CaTPD1_Ch4.2 CaTPD1_Ch4.1 CaTPD1_Ch3 CaTPD1_Ch2 |
| GO:0007186 | G-protein coupled receptor signaling pathway | CaTPD1_Ch4.2 |
| GO:0007267 | cell-cell signaling | CaTPD1_Ch6 |
| GO:0007600 | sensory perception | CaTPD1_Ch2 |
| GO:0007606 | sensory perception of chemical stimulus | CaTPD1_Ch2 |
| GO:0008104 | protein localization | CaTPD1_Ch5 |
| GO:0008380 | RNA splicing | CaTPD1_Ch5 |
| GO:0009059 | macromolecule biosynthetic process | CaTPD1_Ch5 CaTPD1_Ch3 |
| GO:0010468 | regulation of gene expression | CaTPD1_Ch6 CaTPD1_Ch5 CaTPD1_Ch4.1 CaTPD1_Ch3 |
| GO:0016071 | mRNA metabolic process | CaTPD1_Ch5 |
| GO:0019222 | regulation of metabolic process | CaTPD1_Ch6 CaTPD1_Ch5 CaTPD1_Ch4.3 CaTPD1_Ch4.2 CaTPD1_Ch4.1 CaTPD1_Ch3 CaTPD1_Ch2 |
| GO:0031328 | positive regulation of cellular biosynthetic process | CaTPD1_Ch5 |
| GO:0034220 | ion transmembrane transport | CaTPD1_Ch5 |
| GO:0034613 | cellular protein localization | CaTPD1_Ch5 |
| GO:0034645 | cellular macromolecule biosynthetic process | CaTPD1_Ch5 |
| GO:0044255 | cellular lipid metabolic process | CaTPD1_Ch2 |
| GO:0044281 | small molecule metabolic process | CaTPD1_Ch5 |
| GO:0048878 | chemical homeostasis | CaTPD1_Ch3 |
| GO:0050907 | detection of chemical stimulus involved in sensory perception | CaTPD1_Ch2 |
| GO:0051171 | regulation of nitrogen compound metabolic process | CaTPD1_Ch6 CaTPD1_Ch5 CaTPD1_Ch4.2CaTPD1_Ch4.1 CaTPD1_Ch3 |
| GO:0051252 | regulation of RNA metabolic process | CaTPD1_Ch5 |
| GO:0051606 | detection of stimulus | CaTPD1_Ch2 |
| GO:0051641 | cellular localization | CaTPD1_Ch6 CaTPD1_Ch5 CaTPD1_Ch4.3 CaTPD1_Ch4.2CaTPD1_Ch4.1 CaTPD1_Ch3 CaTPD1_Ch2 |
| GO:0051649 | establishment of localization in cell | CaTPD1_Ch6 |
| GO:0055085 | transmembrane transport | CaTPD1_Ch5 |
| GO:1903506 | regulation of nucleic acid-templated transcription | CaTPD1_Ch6 CaTPD1_Ch5 CaTPD1_Ch3 |
| GO:2001141 | regulation of RNA biosynthetic process | CaTPD1_Ch6 |
| **Cellular Component Predictions** | | |
| GO:0005576 | extracellular region | CaTPD1_Ch6 CaTPD1_Ch5 CaTPD1_Ch4.3 CaTPD1_Ch4.2CaTPD1_Ch4.1 CaTPD1_Ch3 CaTPD1_Ch2 |
| GO:0005615 | extracellular space | CaTPD1_Ch6 CaTPD1_Ch4.3 CaTPD1_Ch4.2 CaTPD1_Ch4.1 CaTPD1_Ch3 CaTPD1_Ch2 |
| GO:0005739 | mitochondrion | CaTPD1_Ch5 |
| GO:0005783 | endoplasmic reticulum | CaTPD1_Ch6 CaTPD1_Ch4.3 CaTPD1_Ch4.2 |
| GO:0005886 | plasma membrane | CaTPD1_Ch6 CaTPD1_Ch4.3 CaTPD1_Ch4.2 CaTPD1_Ch4.1 CaTPD1_Ch3 CaTPD1_Ch2 |
| GO:0005887 | integral component of plasma membrane | CaTPD1_Ch6 |
| GO:0009897 | external side of plasma membrane | CaTPD1_Ch6 |
| GO:0009986 | cell surface | CaTPD1_Ch6 CaTPD1_Ch4.3 CaTPD1_Ch4.2 CaTPD1_Ch4.1 CaTPD1_Ch3 |
| GO:0012505 | endomembrane system | CaTPD1_Ch6 CaTPD1_Ch4.3 CaTPD1_Ch4.2 CaTPD1_Ch4.1 CaTPD1_Ch3 CaTPD1_Ch2 |
| GO:0016020 | membrane | CaTPD1_Ch6 CaTPD1_Ch5 CaTPD1_Ch4.3 CaTPD1_Ch4.2CaTPD1_Ch4.1 CaTPD1_Ch3 CaTPD1_Ch2 |
| GO:0016021 | integral component of membrane | CaTPD1_Ch6 CaTPD1_Ch4.2 CaTPD1_Ch4.1 CaTPD1_Ch3 |
| GO:0031090 | organelle membrane | CaTPD1_Ch6 CaTPD1_Ch4.2 CaTPD1_Ch4.1 CaTPD1_Ch3 |
| GO:0031224 | intrinsic component of membrane | CaTPD1_Ch6 CaTPD1_Ch4.2 CaTPD1_Ch4.1 CaTPD1_Ch3 CaTPD1_Ch2 |
| GO:0031982 | vesicle | CaTPD1_Ch6 CaTPD1_Ch4.3 CaTPD1_Ch4.2 CaTPD1_Ch4.1 CaTPD1_Ch3 CaTPD1_Ch2 |
| GO:0031988 | membrane-bounded vesicle | CaTPD1_Ch6 CaTPD1_Ch4.3 CaTPD1_Ch4.2 CaTPD1_Ch4.1 CaTPD1_Ch3 CaTPD1_Ch2 |
| GO:0070062 | extracellular vesicular exosome | CaTPD1_Ch6 CaTPD1_Ch5 CaTPD1_Ch4.3 CaTPD1_Ch4.2 CaTPD1_Ch4.1 CaTPD1_Ch3 CaTPD1_Ch2 |
| GO:0071944 | cell periphery | CaTPD1_Ch6 CaTPD1_Ch5 CaTPD1_Ch4.3 CaTPD1_Ch4.2CaTPD1_Ch4.1 CaTPD1_Ch3 CaTPD1_Ch2 |
| GO:0005576 | extracellular region | CaTPD1_Ch6 CaTPD1_Ch5 CaTPD1_Ch4.3 CaTPD1_Ch4.2 CaTPD1_Ch4.1 CaTPD1_Ch3 CaTPD1_Ch2 |
| GO:0005615 | extracellular space | CaTPD1_Ch6 |
| GO:0005739 | mitochondrion | CaTPD1_Ch5 |
| GO:0005783 | endoplasmic reticulum | CaTPD1_Ch6 |
| GO:0005886 | plasma membrane | CaTPD1_Ch6 |
| GO:0005887 | integral component of plasma membrane | CaTPD1_Ch6 |
| GO:0009897 | external side of plasma membrane | CaTPD1_Ch6 |
| GO:0009986 | cell surface | CaTPD1_Ch6 |
| GO:0012505 | endomembrane system | CaTPD1_Ch6 |
| GO:0016020 | membrane | CaTPD1_Ch6 CaTPD1_Ch5 CaTPD1_Ch4.3 CaTPD1_Ch4.2 CaTPD1_Ch4.1 CaTPD1_Ch3 CaTPD1_Ch2 |
| GO:0016021 | integral component of membrane | CaTPD1_Ch6 CaTPD1_Ch4.2  CaTPD1_Ch4.1 CaTPD1_Ch3 |
| GO:0031090 | organelle membrane | CaTPD1_Ch6 CaTPD1_Ch4.2 CaTPD1_Ch4.1 CaTPD1_Ch3 |
| GO:0031224 | intrinsic component of membrane | CaTPD1_Ch6 CaTPD1_Ch4.2 CaTPD1_Ch4.1 CaTPD1_Ch3 CaTPD1_Ch2 |
| GO:0031982 | vesicle | CaTPD1_Ch6 CaTPD1_Ch4.3 CaTPD1_Ch4.2 CaTPD1_Ch4.1 CaTPD1_Ch3 CaTPD1_Ch2 |
| GO:0031988 | membrane-bounded vesicle | CaTPD1_Ch6 CaTPD1_Ch4.3 CaTPD1_Ch4.2 CaTPD1_Ch4.1 CaTPD1_Ch3 CaTPD1_Ch2 |
| GO:0070062 | extracellular vesicular exosome | CaTPD1_Ch6 CaTPD1_Ch5 CaTPD1_Ch4.3 CaTPD1_Ch4.2 CaTPD1_Ch4.1 CaTPD1_Ch3 CaTPD1_Ch2 |
| GO:0071944 | cell periphery | CaTPD1_Ch6 CaTPD1_Ch5 CaTPD1_Ch4.3 CaTPD1_Ch4.2 CaTPD1_Ch4.1 CaTPD1_Ch3 CaTPD1_Ch2 |
| **Molecular Function Predictions** | | |
| GO:0000166 | nucleotide binding | CaTPD1_Ch5 CaTPD1_Ch4.2 CaTPD1_Ch4.1 |
| GO:0001664 | G-protein coupled receptor binding | CaTPD1_Ch6 |
| GO:0001883 | purine nucleoside binding | CaTPD1_Ch5 |
| GO:0003676 | nucleic acid binding | CaTPD1_Ch5 CaTPD1_Ch4.3 CaTPD1_Ch3 |
| GO:0003677 | DNA binding | CaTPD1_Ch5 |
| GO:0003723 | RNA binding | CaTPD1_Ch5 |
| GO:0003779 | actin binding | CaTPD1_Ch5 |
| GO:0003824 | catalytic activity | CaTPD1_Ch6 CaTPD1_Ch6 CaTPD1_Ch5 CaTPD1_Ch4.3 CaTPD1_Ch4.2 CaTPD1_Ch4.1 CaTPD1_Ch3 CaTPD1_Ch2 |
| GO:0004175 | endopeptidase activity | CaTPD1_Ch4.3 |
| GO:0004857 | enzyme inhibitor activity | CaTPD1_Ch4.2 |
| GO:0004866 | endopeptidase inhibitor activity | CaTPD1_Ch2 |
| GO:0004871 | signal transducer activity | CaTPD1_Ch2 |
| GO:0004888 | transmembrane signaling receptor activity | CaTPD1_Ch4.2 |
| GO:0005125 | cytokine activity | CaTPD1_Ch6 CaTPD1_Ch5 CaTPD1_Ch4.3 CaTPD1_Ch4.2 CaTPD1_Ch4.1 CaTPD1_Ch3 CaTPD1_Ch2 |
| GO:0005126 | cytokine receptor binding | CaTPD1_Ch6 CaTPD1_Ch5 CaTPD1_Ch4.2 CaTPD1_Ch4.1 CaTPD1_Ch3 CaTPD1_Ch2 |
| GO:0005215 | transporter activity | CaTPD1_Ch4.1 CaTPD1_Ch2 |
| GO:0005216 | ion channel activity | CaTPD1_Ch5 |
| GO:0005524 | ATP binding | CaTPD1_Ch4.1 |
| GO:0008083 | growth factor activity | CaTPD1_Ch6 CaTPD1_Ch4.2 CaTPD1_Ch4.1 CaTPD1_Ch3 CaTPD1_Ch2 |
| GO:0008092 | cytoskeletal protein binding | CaTPD1_Ch6 CaTPD1_Ch5 CaTPD1_Ch4.3 |
| GO:0008233 | peptidase activity | CaTPD1_Ch2 |
| GO:0008236 | serine-type peptidase activity | CaTPD1_Ch4.2 CaTPD1_Ch4.1 CaTPD1_Ch2 |
| GO:0008270 | zinc ion binding | CaTPD1_Ch3 |
| GO:0016740 | transferase activity | CaTPD1_Ch5 |
| GO:0016817 | hydrolase activity, acting on acid anhydrides | CaTPD1_Ch5 |
| GO:0016818 | hydrolase activity, acting on acid anhydrides, in phosphorus-containing anhydrides | CaTPD1_Ch5 |
| GO:0030234 | enzyme regulator activity | CaTPD1_Ch5 CaTPD1_Ch2 |
| GO:0030554 | adenyl nucleotide binding | CaTPD1_Ch5 |
| GO:0035639 | purine ribonucleoside triphosphate binding | CaTPD1_Ch5 |
| GO:0038023 | signaling receptor activity | CaTPD1_Ch2 |
